# Predictive value of Dmax and %ΔSUVmax of ^18^F-FDG PET/CT for the prognosis of patients with diffuse large B-cell lymphoma

**DOI:** 10.1186/s12880-023-01138-8

**Published:** 2023-10-31

**Authors:** Jun Dang, Xiaojuan Peng, Ping Wu, Yutang Yao, Xiaofei Tan, Zhenyan Ye, Xuemei Jiang, Xiao Jiang, Yongli Liu, Shirong Chen, Zhuzhong Cheng

**Affiliations:** Department of Nuclear Medicine, Sichuan Clinical Research Center for Cancer, Sichuan Cancer Hospital & Institute, Sichuan Cancer Center, Affiliated Cancer Hospital of University of Electronic Science and Technology of China, Chengdu, China

**Keywords:** Diffuse large B-cell lymphoma, ^18^F-fluoro-deoxyglucose, Positron emission tomography, Prognosis

## Abstract

**Purpose:**

To investigate the prognosis value of a combined model based on ^18^F-fluoro-deoxyglucose positron emission tomography-computed tomography (^18^F-FDG PET-CT) baseline and interim parameters in patients with diffuse large B-cell lymphoma (DLBCL).

**Methods:**

We retrospectively analyzed the PET metabolic parameters and clinical data of 154 DLBCL patients between December 2015 and October 2020. All of these patients underwent ^18^F-FDG PET/CT scan before treatment and after three or four courses of chemotherapy. The optimal cut-off values for quantitative variables were determined by the receiver operating characteristic (ROC) curve. The baseline and interim PET/CT parameters, which respectively included maximum standardized uptake value (SUVmax0), total metabolic tumor volume (TMTV0), standardized total metabolic tumor volume (STMTV0), and the distance between the two furthest lesions (Dmax) and total tumor lesion glycolysis (TTLG1), SUVmax1, TMTV1, and the rate of change of SUVmax (%ΔSUVmax), and clinical characteristics were analyzed by chi-squared test, Kaplan-Meier survival curve, and Cox regression analysis.

**Results:**

Of 154 patients, 35 exhibited disease progression or recurrence. ROC analysis revealed that baseline ^18^F-FDG PET/CT metabolic parameters, including maximum standardized uptake value (SUVmax0), total metabolic tumor volume (TMTV0), standardized total metabolic tumor volume (STMTV0), and the distance between the two furthest lesions (Dmax), along with interim ^18^F-FDG PET/CT metabolic parameters such as total tumor lesion glycolysis (TTLG1), SUVmax1, TMTV1, and the rate of change of SUVmax (%ΔSUVmax), were predictive of relapse or progression in DLBCL patients (P < 0.05). The chi-squared test showed that TMTV0, STMTV0, Dmax, SUVmax1, TMTV1, TTLG1, %ΔSUVmax, Deauville score, IPI, Ann Arbor stage, and LDH were associated with patient prognosis (P < 0.05). Multivariate Cox regression analysis showed that Dmax (P = 0.021) and %ΔSUVmax (P = 0.030) were independent predictors of prognosis in DLBCL patients. There were statistically significant differences in PFS among the three groups with high, intermediate, and low risk according to the combination model (P < 0.001). The combination model presented higher predictive efficacy than single indicators.

**Conclusion:**

The combined model of baseline parameter Dmax and intermediate parameter %ΔSUVmax of ^18^F-FDG PET/CT improved the predictive efficacy of PFS and contributed to the risk stratification of patients, providing a reference for clinical individualization and precision treatment.

Diffuse large B-cell lymphoma, (DLBCL) is one of the most common subtypes of non-Hodgkin’s lymphoma, accounting for 1/3 of all non-Hodgkin’s lymphomas, with genetic mutational heterogeneity. Immunochemotherapy significantly improves the prognosis for most DLBCL patients, but 20–40% fail first-line therapy, leading to an extremely poor prognosis [1]. The prognosis of such patients is expected to be improved if they are screened before treatment and a personalized treatment plan is developed.

As DLBCL high affinity for ^18^F-fluoro-deoxyglucose (^18^F-FDG), the evaluation of efficacy and prognosis by ^18^F-fluoro-deoxyglucose positron emission tomography-computed tomography (^18^F-FDG PET-CT) is a hot research topic in recent years [2]. Studies revealed that ^18^F-FDG PET/CT parameters, including maximum standardized uptake value (SUVmax), metabolic tumor volume (MTV), total lesion glycolysis (TLG), and %ΔSUVmax hold significant prognostic value [3–5]. However, it is still not uniform which parameters should be included in the prognostic criteria, so it is still relevant to further investigate the clinical value of each parameter of PET/CT. Most studies have focused on analyzing single parameters at baseline or midterm, with fewer studies incorporating characteristics reflecting tumor spread. Even fewer studies discuss the prognostic value of combining baseline and midterm PET/CT parameters in DLBCL patients [6, 7]. We analyzed the clinical data and PET/CT baseline and interim parameters of 154 DLBCL patients who received first-line treatment. We aimed to investigate how PET/CT baseline and interim parameters could predict prognosis. This could provide more information about poor prognosis and help clinical treatment.

## Materials and methods

### Clinical data

Retrospectively analyzed the clinical data of 154 patients with DLBCL admitted to Sichuan Cancer Hospital from December 2015 to October 2020. The study has been approved by the hospital ethics committee, the inclusion criteria were as follows: patients with pathologically and immunohistochemically confirmed DLBCL; patients who received firstline CHOP (cyclophosphamide, hydroxydaunomycin, oncovin, and prednisone) chemotherapy or rituximab plus CHOP (R-CHOP); patients all underwent ^18^F-FDG PET/CT scans before and after 3 or 4 cycles of chemotherapy; with complete clinical data. Exclusion criteria: combined history of other tumors; without baseline ^18^F-FDG PET/CT examination or who received antitumor therapy before undergoing baseline ^18^F-FDG PET/CT examination.

### PET/CT imaging

Siemens Biography MCT-64 PET/CT scanning equipment was used for the examination. ^18^F-FDG developer automatically synthesized by Sumitomo cyclotron and a chemical synthesis module, radiochemical purity > 99%. Patients fasting for more than 6 h before the examination, with blood glucose < 11.1mmol/L after injection of ^18^F-FDG at 4.0 MBq/kg body mass, the patients were instructed to lie still for 1 h. PET/CT imaging was performed after urination, with the acquisition range from the cranial vault to the mid-femur, and 6 to 7 beds were acquired. Prior CT scan: tube voltage 140 kV, effective current 42 mAs, pitch 0.8, spherical tube single-turn rotation time 0.5 s, layer thickness 8 mm. PET scans were acquired in 3D, 1.5 min/bed, with delayed imaging, if necessary. Images were reconstructed using the ordered subsets expectation maximization (OSEM) iterative algorithm, and image fusion and post-processing were performed on a Siemens MMWP workstation.

### Image analysis

PET image data in anonymized Digital Imaging and Communications in Medicine (DICOM) format were collected for functional parameter measurements using LIFEx software [8]. Lesions were defined as areas with increased uptake of ^18^F-FDG on PET and abnormal density on CT. Two experienced physicians then reviewed the resulting clusters to remove physiological uptake based on interim PET/CT results. Finally, Dmax, SUVmax, TMTV, and TTLG are automatically generated. MTV was calculated based on a supervised segmentation of tumor regions involving 41% SUVmax thresholding of automatically detected hypermetabolic regions. The dissemination feature Dmax was defined as the distance between the two lesions that were the furthest apart. Each lesion location was defined as the position of its center, and the distances between two lesions were calculated using the Euclidian distance between their centers. TMTV was defined as the sum of every individual lesion’s metabolic volume. TTLG was obtained by summing the tumor lesion glycolysis over all lesions. Other parameters such as %ΔSUVmax= (baseline SUVmax - interim SUVmax) / baseline SUVmax. %ΔTMTV, %ΔTTLG were calculated as above. Standardized total tumor metabolic volume pair (STMTV) = TMTV/weight, standardized total tumor lesion glycolysis (STTLG0) as above. The Deauville score was defined as positive if it was ≥ 4 and negative if it was < 4.

### Follow-up assessment

All patients were followed up by telephone or outpatient visits, dated through December 31, 2021. Progression-free survival (PFS) was the clinical endpoint of this retrospective study, which refers to the time from diagnosis to disease recurrence, progression, or final follow-up.

### Statistical analysis

SPSS 26.0 software and GraphPad Prism 9 were used for statistical analysis and measured data within a normal distribution are expressed as the mean ± standard deviation (SD), those not conforming are expressed as median (upper and lower quartiles). The receiver operating characteristics (ROC) curves were used to determine the optimal cutoff values for SUVmax, TMTV0, STMTV0, TTLG0, STTLG0, Dmax, SUVmax1, TMTV1, TTLG1, %ΔSUVmax, %ΔTMTV, and %ΔTTLG and performed to evaluate the predictive efficacy of the indicators. Comparison between groups by chi-squared test and the meaningful parameters were included in the multivariate Cox regression analyses. A Kaplan-Meier (K-M) survival analysis was used to complete the survival analysis. Statistical significance was defined as a P value less than 0.05.

## Results

The study included 154 patients with DLBCL available for evaluation and analysis. Among them, 78 cases were males and 76 cases were females. The median age was 56 (43, 65) years and the range was 16–87 years. Table 1 shows the clinical characteristics of the 154 DLBCL patients. The median follow-up time was 28 months, with a range of 3.0 to 73.5 months. There were 35 cases of disease progression at the time of follow-up to date.

**Table 1:** Clinical and imaging characteristics

Characteristics	n = 154(%)	Progress or recurrence(%)
Age	56(43,65)	
Range	16–87	
Sex		
Male	78(51%)	22(28%)
Female	76(49%)	13(27%)
Ann Arbor stage		
I	4(3%)	0(0%)
II	52(34%)	4(8%)
III	32(21%)	6(19%)
IV	66(43%)	25(38%)
LDH level		
Normal	94(61%)	14(15%)
Abnormal	60(39%)	21(35%)
IPI score		
0	30(19%)	2(7%)
1	41(27%)	5(12%)
2	40(26%)	9(23%)
3	35(23%)	13(37%)
4	7(5%)	5(71%)
5	1(1%)	1(100%)
SUVmax0	23.29 ± 9.43	
Deauville		
1	38(25%)	7(18%)
2	35(23%)	5(14%)
3	31(20%)	4(13%)
4	19(12%)	5(26%)
5	31(20%)	14(45%)

LDH, lactate dehydrogenase; IPI, International prognostic index; SUVmax, maximum standardized uptake value

Of the 154 patients, 61 cases were ≥ 60 years and 93 cases were < 60 years; 56 cases in Ann Arbor stage I-II and 98 cases in stage III-IV; the international prognostic index (IPI) was > 2 points in 43 cases and ≤ 2 points in 111 cases; the lactate dehydrogenase (LDH) level was normal in 94 cases and abnormal in 60 cases. Among them, patients with Ann Arbor stage III-IV (P < 0.001), IPI > 2 points (P < 0.001), and abnormal LDH (P = 0.004) had a higher risk of disease progression or recurrence (Table 2).

**Table 2:** The effects of clinical characteristics on disease progression or recurrence

Characteristics	No. of patients	No relapse	Progress or recurrence	χ2	*P*
Sex					
Male	78	56	22	2.710	0.100
Female	76	63	13		
Age (years)					
≤ 60	93	77	16	4.078	0.043
>60	61	42	19		
Ann Arbor stage					
I+II	56	52	4	10.815	<0.001
III+IV	98	67	31		
LDH level					
Normal	94	80	14	8.430	0.004
Abnormal	60	39	21		
IPI score					
≤ 2	71	64	7	12.421	<0.001
>2	83	55	28		

LDH, lactate dehydrogenase; IPI, International prognostic index

We defined AUC > 0.6 as having diagnostic value and performed ROC analysis. The results showed that the cutoff values of TMTV0, STMTV0, Dmax, SUVmax1, TMTV1, TTLG1, and %ΔSUVmax for PFS were 152.11, 2.63, 53.20, 5.31, 30.03, 78.97, and 87.82%, respectively (Table 3). Of these metabolic parameters, patients with TMTV0, STMTV0, Dmax, SUVmax1, TMTV1, TTLG1 above the cutoff (P < 0.05), %ΔSUVmax below the cutoff (P < 0.05) and Deauville score ≥ 4 points (P < 0.05) had a higher risk of disease progression or recurrence (Table 4).

**Table 3:** ROC analysis of PET parameters to PFS

Variables	AUC	*P*	cutoff value	Sensitivity (%)	Specificity (%)
SUVmax0	0.483	0.758	28.07	77.1	34.5
TMTV0	0.618	0.035	152.11	82.9	46.2
STMTV0	0.619	0.033	2.63	82.9	47.9
TTLG0	0.585	0.127	799.63	82.9	41.2
STTLG0	0.580	0.149	13.38	82.9	39.5
Dmax	0.707	< 0.001	53.20	51.4	82.4
SUVmax1	0.664	0.003	5.31	48.6	86.6
TMTV1	0.628	0.021	30.03	28.6	95.8
TTLG1	0.649	0.008	78.97	34.3	93.3
%ΔSUVmax	0.686	0.001	87.82	77.1	57.1
%ΔTMTV	0.572	0.195	95.37	42.9	78.2
%ΔTTLG	0.594	0.092	98.41	40.0	79.8

AUC, area under the receiver operating characteristic curve; SUVmax, maximum standardized uptake value; MTV, metabolic tumor volume; TLG, total lesion glycolysis; Dmax, defined as the distance between the two lesions that were the furthest apart. The subscripts 0 and 1 represent baseline and interim measures, respectively

**Table 4:** The effects of metabolic parameters on disease progression or recurrence

parameters	No. of patients	No relapse	Progress or recurrence	χ2	P
TMTV0(cm^3^)					
<152.11	61	55	6	9.558	0.002
≥ 152.11	93	64	29		
STMTV0(cm^3^)					
<2.63	63	57	6	10.583	0.001
≥ 2.63	91	62	29		
Dmax(mm)					
<53.20	115	98	17	16.320	< 0.001
≥ 53.20	39	21	18		
SUVmax1					
<5.31	121	103	18	19.820	< 0.001
≥ 5.31	33	16	17		
TMTV1(cm^3^)					
<30.03	139	114	25	18.270	< 0.001
≥ 30.03	15	5	10		
TTLG1(g)					
<78.97	133	110	23	16.399	< 0.001
≥ 78.97	21	9	12		
%ΔSUVmax					
<87.82%	77	51	26	10.686	0.001
≥ 87.82%	77	68	9		
Deauville					
<4	104	88	16	9.834	0.002
≥ 4	50	31	19		

SUVmax, maximum standardized uptake value; MTV, metabolic tumor volume; TLG, total lesion glycolysis; Dmax, defined as the distance between the two lesions that were the furthest apart. The subscripts 0 and 1 represent baseline and interim measures, respectively

**Fig. 1:**
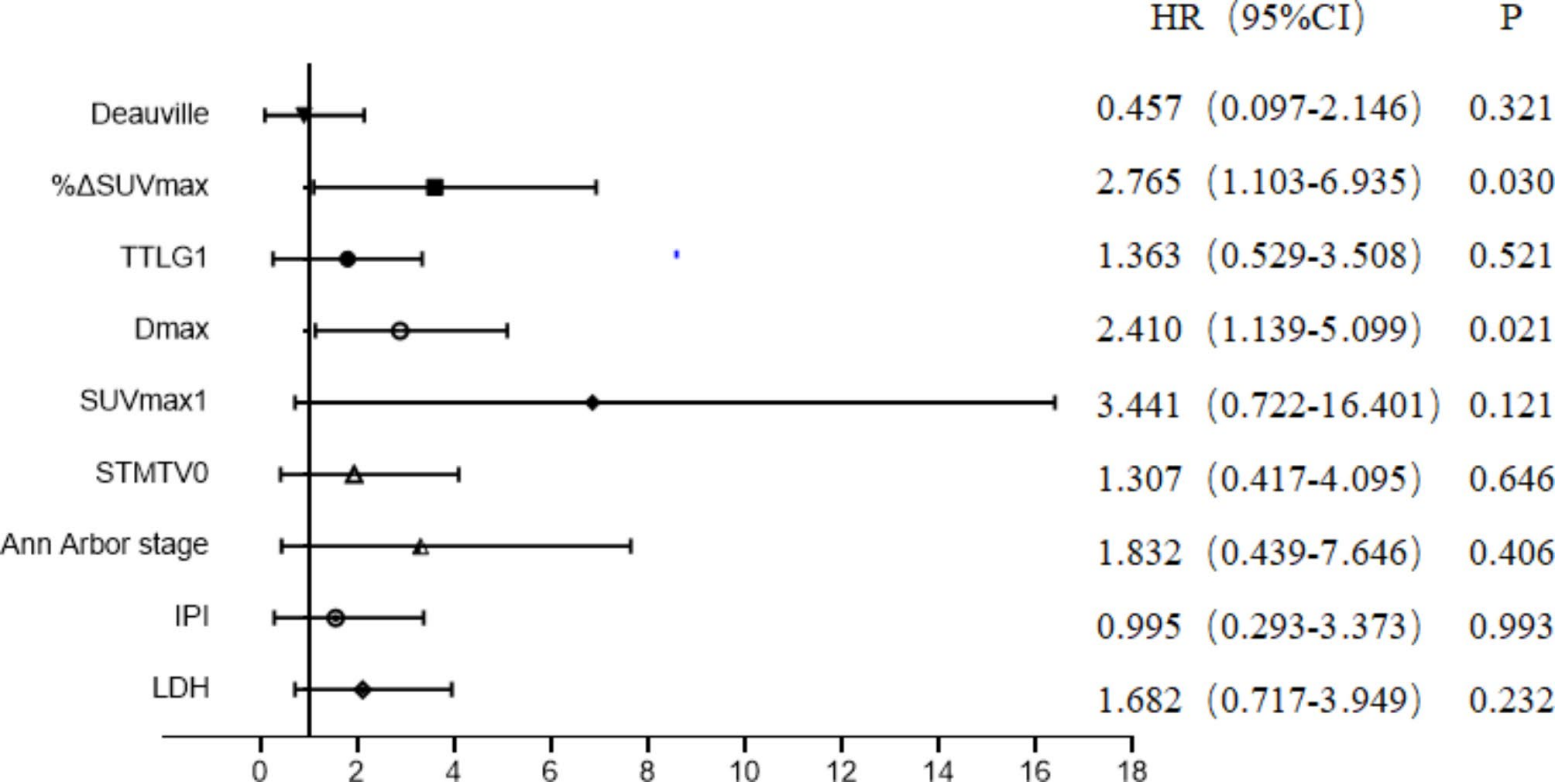
Multivariate Cox regression analyses of clinical characteristics, baseline and intermediate PET/CT parameters for PFS, and Comparison of 95% confidence intervals for various parameters

**Fig. 2:**
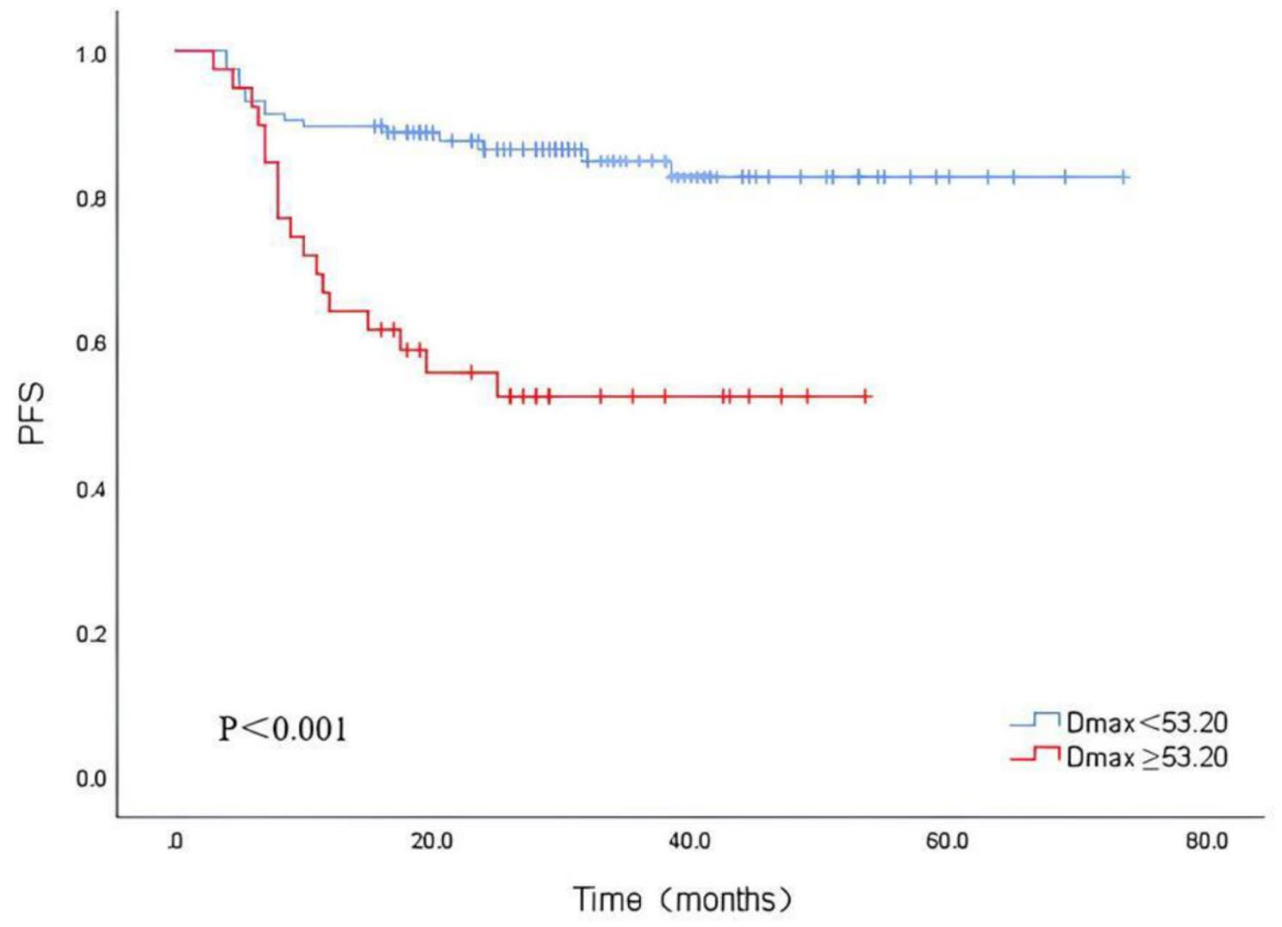
Kaplan‑Meier survival analysis of PFS according to Dmax

**Fig. 3:**
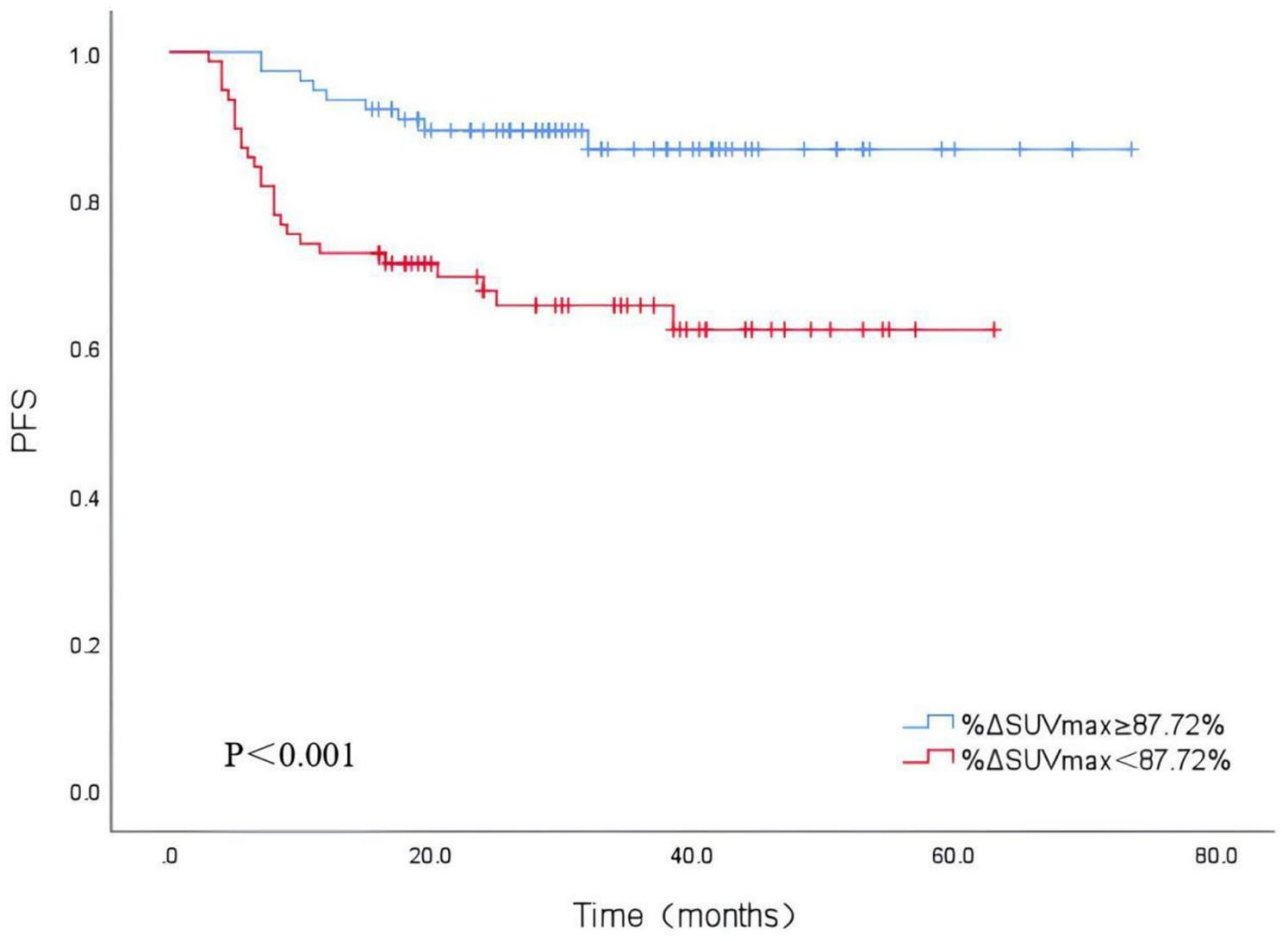
Kaplan‑Meier survival analysis of PFS according to %ΔSUVmax

The parameters that were meaningful in the chi-squared test were included in the multivariate cox regression analyse. Due to the close correlation between TMTV0 and STMTV0, TMTV1 and TTLG1, only STMTV0 and TTLG1 were included in the multivariate analysis. The analysis indicated that %ΔSUVmax (HR = 2.765, 95% CI = 1.103–6.935, P = 0.030) and Dmax (HR = 2.410, 95% CI = 1.139–5.099, P = 0.021) were independent risk factors for PFS in patients with DLBCL (Fig. 1). Kaplan-Meier survival curve analysis showed that PFS was better in the group with Dmax < 53.20 cm than in the group with Dmax ≥ 53.20 cm (Fig. 2); PFS was better in the group with %ΔSUVmax ≥ 87.82% than in the group with %ΔSUVmax < 87.82% (Fig. 3), suggesting that patients with high Dmax before treatment and low %ΔSUVmax after treatment had a poorer prognosis and were more likely to recur or progress.

**Fig. 4:**
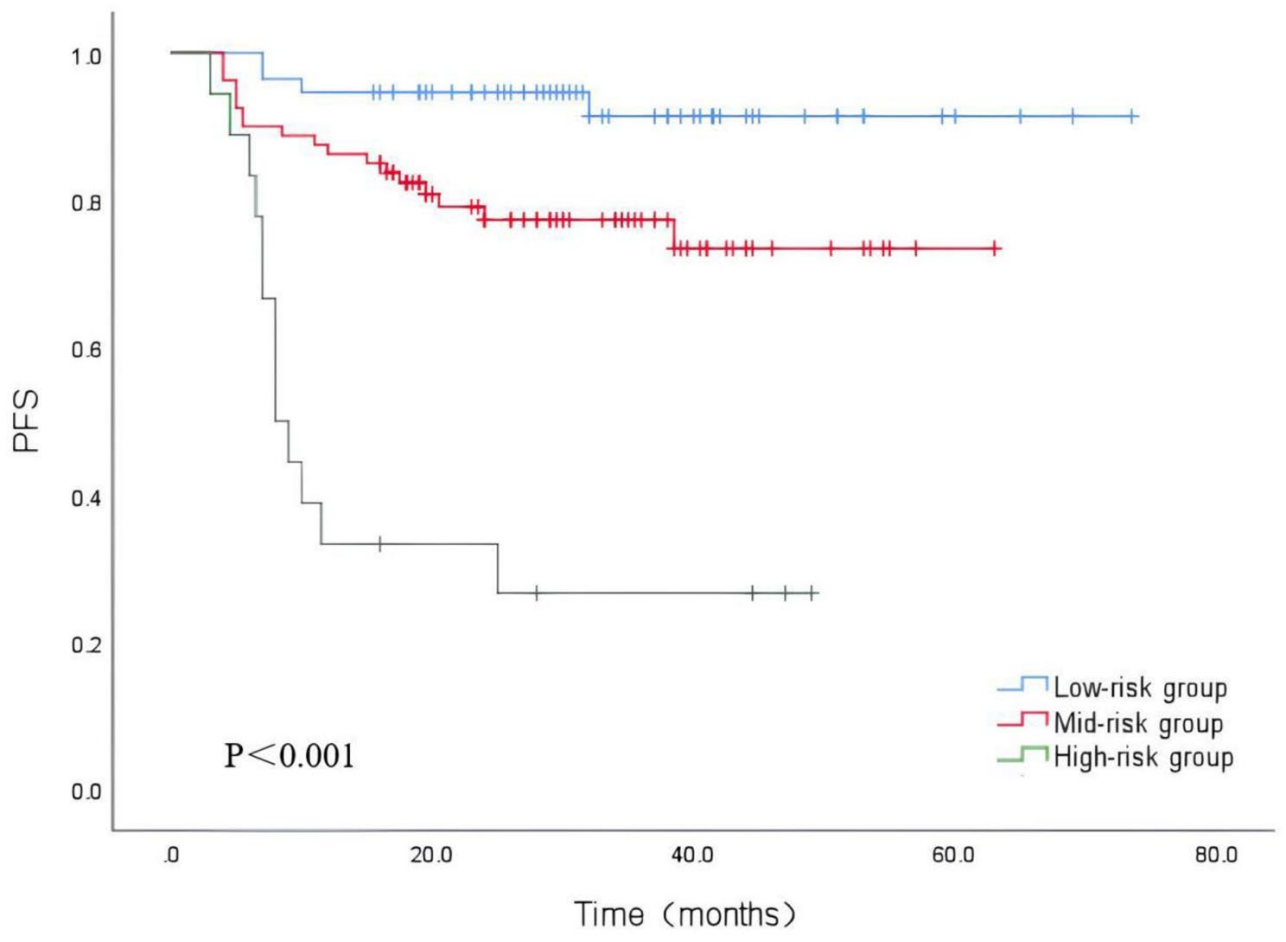
Kaplan‑Meier survival analysis of PFS according to the Combination Model

A new model was established by combining the parameters of Dmax and %ΔSUVmax, and patients were divided into three groups: the high-risk group was Dmax ≥ 53.20+%ΔSUVmax < 87.82%; the low-risk group was Dmax < 53.20+%ΔSUVmax ≥ 87.82%; and the remaining combination was the medium-risk group. Kaplan-Meier survival curve showed that PFS was statistically different between all three groups (P < 0.001), PFS in the low-risk group was significantly higher than in the medium- and high-risk groups (Fig. 4).

**Fig. 5:**
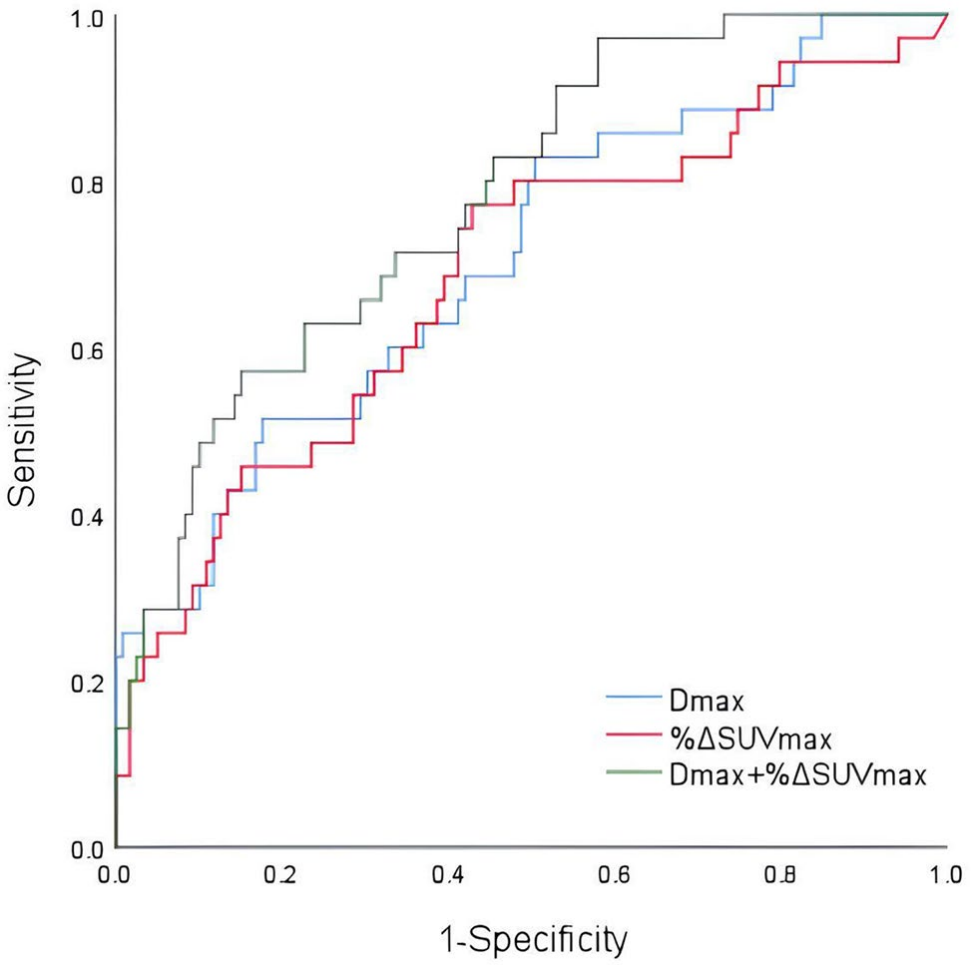
ROC curves of Dmax and %ΔSUVmaxand the Combination Model for prediction of PFS

According to the ROC of Dmax, %ΔSUVmax and the combination of both to PFS, the AUCs, sensitivities and specificities were 0.707, 51.4%, 82.4%, 0.686, 77.1%, 57.1% and 0.779, 57.1%, 84.9%, respectively. The combined model improves the predictive performance compared to single parameters (Fig. 5).

## Discussion

DLBCL is a clinically and pathologically heterogeneous disease, which poses a challenge for determining treatment efficacy and prognosis. Hence, the prognostic factors in DLBCL have been a hot topic of research. The IPI score is a clinical index often used to determine the prognosis of non-Hodgkin’s lymphoma, especially DLBCL. As rituximab therapy becomes available, the IPI has also been improved by introducing variants such as R-IPI, aa-IPI and NCCN-IPI, which can better reflect the prognosis of DLBCL patients with different chemotherapy regimens [9]. In the present study, there was a significant difference in PFS between the low and high IPI subgroups, which is consistent with the findings of previous studies, suggesting that IPI has important value in prognostic assessment. However, the multifactorial analysis indicated that IPI was not an independent predictor, similar to previous results [7, 10, 11]. This study also showed that the prognosis could vary among patients with the same IPI score. The IPI score was based on the patient’s pretreatment status and did not incorporate the patient’s treatment response to chemotherapy, so its use as a prognostic evaluation index has some limitations. In addition, Ann Arbor stage and LDH levels were also associated with prognosis, but they were not independent predictors.

The imaging agent ^18^F-FDG of PET/CT can accumulate in tumour cells with increased metabolism and proliferation, thus indicating lesion activity more accurately than conventional imaging [12]. Therefore, baseline and interim ^18^F-FDG PET/CT parameters are widely used to study the prognosis of DLBCL patients. In our study, Dmax was the only independent predictor of PFS among all baseline parameters, and the risk of disease progression in patients with high Dmax was 1.410 times higher than that in the low-value group. Patients with Dmax ≥ 53.20 cm had significantly lower PFS than those with Dmax < 53.20 cm, indicating that high Dmax was associated with poor prognosis. This result was similar to Zhou et al.‘s finding (57.4 cm) [13]. Cottereau’s study [4] also led to the conclusion that high Dmax was associated with poor prognosis and indicated that there was no significant difference in Dmax among patients with different heights. In addition, baseline parameters such as TMTV0 and STMTV0, although not independent predictors, were all associated with patient PFS. This is further evidence that baseline metabolic parameters are valuable for prognostic prediction. Some previous studies [14–16] found that pretreatment TMTV was an independent risk factor for prognosis in DLBCL patients. Some studies [6, 17, 18] have also conclusively indicated that TTLG is an independent predictor of prognosis. Our findings are not fully consistent with the abovementioned studies, which may be due to the inconsistent methods of outlining TMTV and the different survival endpoints selected. For example, in MIKHAEELNG [16], a fixed threshold method with SUV = 2.5 was used as the absolute limit, and all metabolic regions > 2.5 were included in the metabolic volume, yet the results were often higher than the true level for patients with high background metabolic levels as well as low overall tumour metabolic levels. In this study, the percentage threshold method was used, and the 41% recommended by the EANM guidelines [19] was chosen as the outline threshold. However, when the tumour SUVmax is too large, the outlined TMTV will underestimate the actual tumour volume and vice versa. Weiler-Sagie [2] analysed 766 DLBCL patients with ^18^F-FDG uptake before chemotherapy and found that more than 97% of lesions exhibited high uptake of ^18^F-FDG. Therefore, there are many interfering factors in the outline of TMTV, while Dmax, as the distance between the centres of the two most distant lesions, can be used to visualize the spatial distribution of the disease, which is not highly dependent on the lesion contour and is not seriously affected by PET/CT instrument performance and image outline, promoting its widespread use.

Compared to baseline parameters, interim ^18^F-FDG PET/CT metabolic parameters can reflect tumour sensitivity to first-line treatments such as R-CHOP, thereby identifying patients who are not sensitive to first-line treatment regimens and guiding clinical changes to improve prognosis. The Deauville score [20] is a widely used clinical method to assess the efficacy of lymphoma by interim ^18^F-FDG PET/CT. It measures the SUVmax of the lesion and compares it with the SUVmax of the liver and mediastinal blood pool on the current imaging. The present study showed poorer PFS in patients with an interim PET/CT Deauville score ≥ 4, which is consistent with the results of previous studies [9]. However, in this study, %ΔSUVmax was the only independent predictor among all interim metabolic parameters and the Deauville score. Patients with low %ΔSUVmax had a 1.765 times higher risk of disease progression than those with high %ΔSUVmax. The results showed that PFS was significantly higher in patients with %ΔSUVmax ≥ 87.82% than in patients with %ΔSUVmax < 87.82%, similar to the results of Zhang et al. [6] (86.02%). Casasnovas et al. [5] also found that %ΔSUVmax predicted PFS in patients after chemotherapy and found better agreement by comparing three readers using %ΔSUVmax and Deauville score to assess efficacy. Rekowski et al. [21] also concluded by comparing the two that %ΔSUVmax seems to be more appropriate to assess the early metabolic response of DLBCL patients to standard R-CHOP treatment. This result may be explained by two reasons. First, the Deauville score was used to select the SUVmax of the lesion and a comparison was made with the SUVmax of the liver and mediastinal blood pool, but this only reflected the metabolism of the local tumour tissue, not the systemic tumour load. Second, factors such as blood glucose, lipids, and age [22] may confound the SUVmax values of the liver and mediastinal blood pool. In contrast, %ΔSUVmax is a semiquantitative parameter used in mid-term PET/CT imaging which is easy to calculate and can reflect the metabolic level of the tumour more objectively. However, the optimal cutoff values of %ΔSUVmax have been reported differently; for example, some scholars [5, 23, 24] reported cutoff values of 70%, 74%, 81.54%, etc., which may be related to blood glucose levels, selection of target lesions, and different instrument specifications. However, Wang et al. [25] found that the Deauville score and %ΔSUVmax were associated with the prognosis of DLBCL patients, but only the Deauville score was an independent predictor. Ng et al. [26] also showed that compared to %ΔSUVmax, the Deauville score was able to better discriminate the prognosis of DLBCL patients. There are some differences between the results of the above studies and those of the present study, which can be explained as follows: (1) In some studies, time-to-progression (TTP) was chosne as the follow-up endpoint, while the endpoint of our study was PFS; (2) The present study included patients with 3–4 cycles of postchemotherapy, while some studies included patients with 2 or 4 cycles of postchemotherapy; (3) The present study included a large number of clinical and imaging parameters, which might influence each other; (4) Meignan et al. [27] pointed out that %ΔSUVmax may be a false-positive in the condition of a low SUVmax level before treatment. Therefore, whether %ΔSUVmax can replace the Deauville score still needs to be confirmed by large-sample, multicentre studies. In addition, the present study showed that the midterm parameters SUVmax1, TMTV1, and TTLG1 were all associated with patient PFS, similar to the results of previous studies, suggesting that the interim metabolic parameters also have good predictive value for prognosis. We extended the previous studies of baseline combined with interim parameters [6, 7] by adding SMTV0, STLG0, %ΔTMTV and %ΔTTLG as potential prognostic predictors.

Out of 77 patients in this study who had a %ΔSUVmax ≥ 87.82%, indicating a good interim treatment response, 9 patients still experienced relapse or progression. Therefore, judging the prognosis based only on the interim response to chemotherapy in clinical practice is not sufficient. We aimed to develop a combined model that integrated ^18^F-FDG PET/CT baseline and interim metabolic parameters which could enhance predictive efficacy and identify high-risk patients. We propose a prognostic assessment model that uses these two complementary parameters from baseline and interim PET/CT scans to characterize two distinct aspects of the disease: tumour dissemination and posttreatment response. This study showed that the predictive efficacy of the combined Dmax+%ΔSUVmax was higher than that of the single parameter, and the PFS of patients in all three combined models was significantly different, with medium- and high-risk patients having significantly lower PFS than low-risk patients. Cottereau [28] suggested that combining both Dmax and MTV could further improve the risk stratification of patients. Zhang et al. [6] combined both baseline TLG and %ΔSUVmax and showed good predictive power for recurrence or progression. Zhu et al. [7] suggested that combining the maximum diameter of the largest lesion and midterm treatment response could improve the efficacy of predicting PFS and help identify patients at high risk of recurrence. Few studies have combined baseline and intermediate metabolic parameters to build a combined model to predict prognosis, and the indicators used vary, but all suggest that PET/CT baseline and intermediate parameters should be used as a reference for patient risk stratification, thus aiding in the detection of high-risk patients and guiding clinical personalized treatment.

Due to insufficient follow-up time, only PFS was observed in this study, and adequate overall survival was not yet observed, pending continued long-term follow-up to enrich the data. In addition, this study is a retrospective study, and a prospective study is feasible at a later stage to validate the findings.

In conclusion, combining ^18^F-FDG PET/CT baseline and interim metabolic parameters and even further including clinical and pathological indicators to establish a combined model to comprehensively assess patient prognosis may be a future research direction. By combining Dmax, which can reflect lesion dissemination, and %ΔSUVmax, which can indicate treatment response, the predictive efficacy of PFS can be improved, and the risk stratification of patients can be facilitated. This can provide a basis for clinical individualization and precision treatment.

## Data Availability

The datasets presented in this article are not readily available. Requests to access the datasets should be directed to dangjun0913@163.com.
